# Accuracy and the role of experience in dynamic computer guided dental implant surgery: An *in-vitro* study

**DOI:** 10.4317/medoral.22785

**Published:** 2018-12-24

**Authors:** Adrià Jorba-García, Rui Figueiredo, Albert González-Barnadas, Octavi Camps-Font, Eduard Valmaseda-Castellón

**Affiliations:** 1 DDS. Faculty of Medicine and Health Sciences of the University of Barcelona (Spain); 2 DDS, MS, PhD. Associate Professor of Oral Surgery. Coordinator of the Master’s degree program in Oral Surgery and Implantology. Faculty of Medicine and Health Sciences of the University of Barcelona (Spain). Researcher at the IDIBELL Institute, Barcelona (Spain); 3 DDS, MS. Faculty of Medicine and Health Sciences of the University of Barcelona (Spain); 4 DDS, MS. Associate Professor of Oral Surgery. Faculty of Medicine and Health Sciences of the University of Barcelona (Spain). Researcher at the IDIBELL Institute, Barcelona (Spain); 5 DDS, MS, PhD. Professor of Oral Surgery. Director of the Master’s degree program in Oral Surgery and Implantology. Faculty of Medicine and Health Sciences of the University of Barcelona (Spain). Researcher at the IDIBELL Institute, Barcelona (Spain)

## Abstract

**Background:**

To compare the accuracy of implant placement using the conventional freehand method and a dynamic navigation system; to assess the role of the surgeon’s experience in implant placement using these two methods.

**Material and Methods:**

A randomized *in-vitro*study was conducted. Six resin mandible models and 36 implants were used. Two researchers with differing clinical experience (novice and experienced) placed implants using either the Navident dynamic navigation system (navigation group) or the conventional freehand method (freehand group). Accuracy was measured by overlaying the real position in the postoperative CBCT on the virtual presurgical placement of the implant in a CBCT image. Descriptive and bivariate analyses of the data were performed.

**Results:**

The navigation group showed significantly higher accuracy for all the variables studied except 3D entry and depth deviation. This system significantly enhanced the accuracy of the novice professional in several outcome variables in comparison with the freehand implant placement method. However, when the implants were placed by the experienced clinician the dynamic navigation system only improved angulation deviation. Significant differences were found between the 2 professionals when the freehand method was employed. Similar deviations were observed for the implants placed with the navigation system.

**Conclusions:**

Dynamic computer assisted surgery systems allow more accurate implant placement in comparison with the conventional freehand method, regardless of the surgeon’s experience. However, this system seems to offer more advantages to novice professionals, since it allows them to reduce their deviations significantly and achieve similar results to those of experienced clinicians.

** Key words:**Computer guided surgery, dynamic computer guided surgery, implant navigation system, dental implants.

## Introduction

Nowadays, dental implants have high success rates and are considered to be a reliable treatment option to rehabilitate both partially and totally edentulous patients (1,2). Adequate surgical diagnosis is paramount, to avoid complications and damage to important anatomical structures, to facilitate the prosthetic treatment and to evaluate the quality and quantity of available bone (3-7).

The development of new imaging technologies like Cone-Beam Computer Tomography (CBCT) has led to a great advance in presurgical planning in comparison with panoramic radiographies, since it provides three-dimensional (3D) data about the patient’s anatomy (8,9). In addition, it is now possible to place the dental implants in their ideal position virtually, through various software programs, using the data provided by CBCT scans (3,10).

Several methods based on Computer Assisted Surgery (CAS) that aim to minimize the differences between the preoperative planning and the final treatment outcome have been described. CAS methods can be considered static when stereolithographic templates are employed during the drilling and the insertion of the dental implant, or dynamic when an intraoperative real-time tracking device is used to monitor whether the drills and implants are following the planned insertion path (3,6,7,9,10).

**Figure 1:**
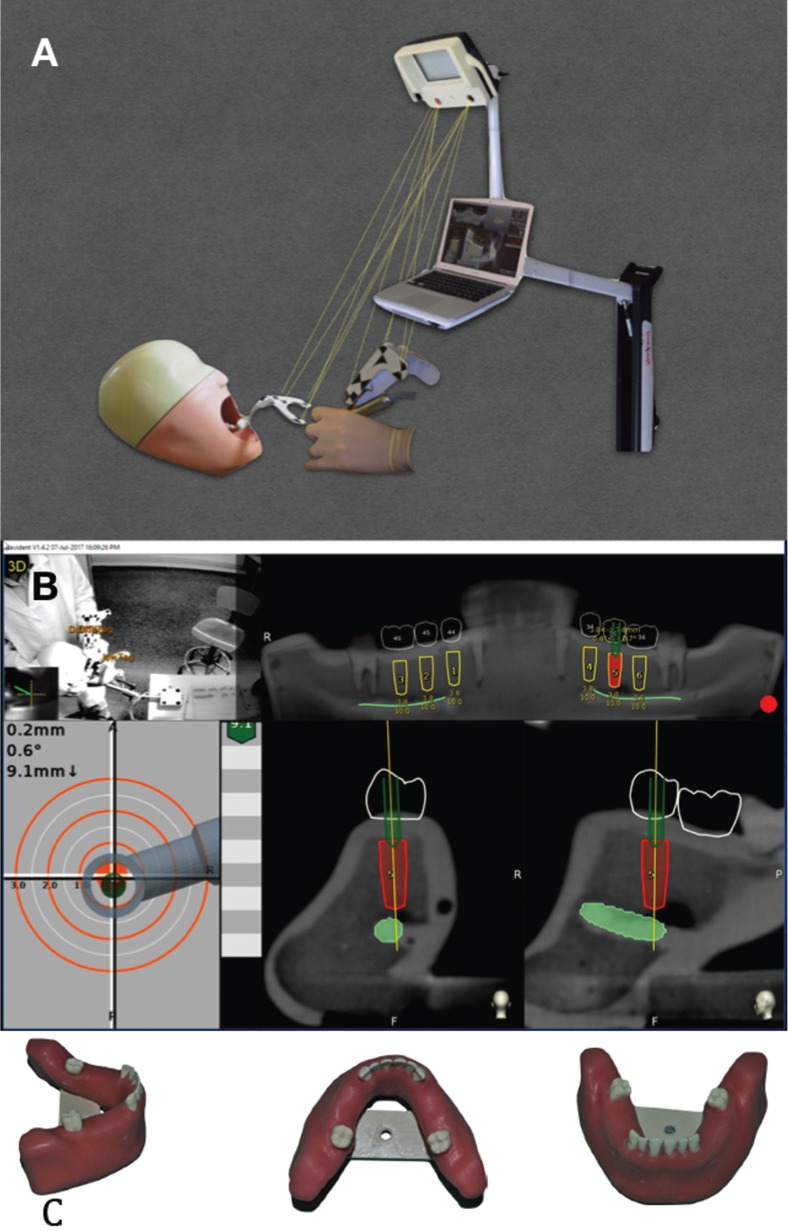
Navident: A: Navident© work diagram; B: Navident© software interface during surgical procedures; C: Artificial resin models employed in the study.

Dynamic computer assisted surgery, also known as a surgical navigation system or guided surgery system, makes it possible to determine the real position of the surgical drill on the reconstructed 3D image provided by CBCT. It guides the surgeon to the position planned preoperatively while performing the surgical procedure (Fig. 1 A,B) (6,9).

Several studies have been published about dynamic computer assisted surgery systems and their high accuracy has been proven and assessed. It has been shown that sinus perforations or inferior alveolar nerve injuries during drilling can be reduced by using these guided systems (5,11).

The use of computer guided surgery is usually indicated for complex cases in which anatomic situations, such as the proximity of the inferior alveolar nerve, make very accurate surgery necessary in order to avoid injures. Hence, a knowledge of the maximal possible deviation of these systems is very relevant for daily clinical practice (9). Moreover, to the best of the authors’ knowledge, no data have been published on the role of the surgeon’s experience in the use of such technology.

Therefore, the aims of this study were to assess the accuracy of implant placement using a dynamic navigation system compared with the conventional freehand technique, and whether implant placement accuracy differed between novice and experienced professionals using these two methods.

## Material and Methods

A randomized *in-vitro* study was conducted to compare implant placement with the dynamic navigation system Navident® (Navident®, ClaroNav Technology Inc.®, Toronto, Canada) and with the conventional freehand technique. The CONSORT guidelines were followed throughout the study (12).

**Figure 2:**
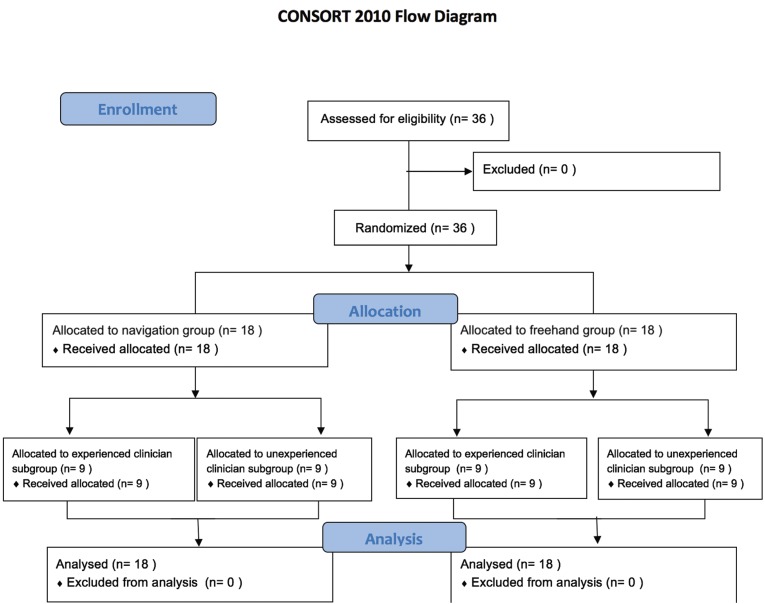
Consort flow diagram showing allocation to the participants in the study.

Two researchers placed 36 dental implants (Ticare InHex standard 3.75mm x 10mm; MG Mozo-Grau®, Valladolid, Spain) in 6 partially edentulous mandible models (BoneModels®, Castellón de la Plana, Spain) specifically designed for this study (Fig. 1C). The models were made using exactly the same template and were missing 3 adjacent teeth on both sides (first and second premolar and first molar). A radio-opacifier was employed in relevant structures like the adjacent teeth and the inferior alveolar canal (Fig. 1B). The 2 researchers had different degrees of experience: One (AJG) was a final year undergraduate dental degree student at the University of Barcelona, with no experience in implant dentistry, while the other (RF) was an experienced oral surgeon (over 15 years of clinical experience in implant dentistry) (Fig. 2).

The sample size was calculated with G*Power v.3.1.3 (Heinrich-Heine Universität, Düsseldorf, Germany), taking into account that the primary outcome variable was angular deviation. Mean angulation deviation data were extracted from a previously published study (13). An alpha value of 0.05 and a statistical power of 90% were established. The sample size calculation resulted in 36 implants being considered necessary for this study (18 implants for each group).

Before placing the dental implants, a splint (NaviStent) was firmly attached to the remaining anterior teeth of the mandible. Fiducial markers were attached to the splint and CBCT scans (Planmeca ProMax® 3D Mid (Planmeca, Helsinki, Finland) of all the models were made with the following setup: 90Kv, 10mA, 13.9 seconds, 1245 DAP (mGy*cm2), 0,4mm Voxel).

CBCT DICOM data were uploaded to the navigation system software (Navident®) and its planning utilities were used to define the dental arch, inferior alveolar nerve path and position of each implant on the CBCT images. Implants were placed virtually in the first and second premolar and first molar positions on each side, taking into account the most suitable position for the final restoration (Fig. 1B). Prosthetic crowns were also drawn on the CBCT image. To minimize bias, during this phase the surgeons were blinded regarding the group to which each implant was assigned.

The models were randomly allocated to the two researchers, each of whom placed 18 implants in three models in teeth positions 3,4, 3,5, 3,6 and 4,4, 4,5, 4,6. Each implant site was assigned to the navigation system or the freehand system using a website generated random sequence (www.randomization.com). In order to guarantee allocation concealment, the researchers were not told which group each implant was assigned to until just before starting the drilling sequence (after raising the flap). The allocation ratio was 1:1.

Each model was placed in a preclinical learning dental simulator with limited mouth opening and with a latex face to limit visibility and to mimic facial soft tissues. The setting used for the study was very similar to a real clinical scenario in an ergonomic position.

A crestal incision was made with a type 15C scalpel blade. Soft tissue was detached with a Freer elevator. Drilling was then performed while separating the soft tissues with a Minnesota retractor.

The surgeons used the recommended drilling protocol for Ticare InHex standard 3,75mm dental implants (MG Mozo Grau SA, Valladolid, Spain). In the navigation system group, drill axis and tip calibration were performed before starting drilling with a new bur and repeated before implant insertion. The implants were placed with an implant carrier and specific burs at 15 rpm with a maximum torque of 50 N.cm.

To assess the accuracy of both methods, a second CBCT (Planmeca ProMax® 3D Mid (Planmeca, Helsinki, Finland) scan of each model was performed after implant placement, using the following setup: 90Kv, 10mA, 13.9 seconds, 1245 DAP (mGy*cm2), 0,4mm Voxel). A third independent, blinded researcher (AGB) then overlaid the preoperative and postoperative CBCT scans, using the EvaluNav® software (ClaroNav Technology Inc. ®, Toronto, Canada), and compared the planned position with the final position of the dental implant.

For each implant placed, the ideal position of the fixture according to the presurgical virtual planning was compared with the real final position of the implant, measuring the following variables: entry three-dimensional (3D) deviation (3D deviation in the coronal aspect of the alveolar ridge), entry two-dimensional (2D) deviation (2D deviation in the coronal aspect of the alveolar ridge), apex 3D deviation (3D deviation in the apical area of the implant), apex depth deviation (vertical deviation) and angular deviation.

In order to guarantee unbiased statistical analysis, the group (freehand or navigation) and operator (experienced or novice) variables were coded and a blinded researcher (OCF) analyzed the data using Statistical Package for the Social Sciences software (SPSS version 22.0; Armonk, NY, USA: IBM Corp.). The level of significance for all the statistical tests was set at 5% (*p* <0.05).

The normality of the scale variables (entry 3D, entry 2D, apex 3D, apex vertical, angulation and surgical time) was explored using the Shapiro-Wilks test and visual analysis of normal P-P graphics and box diagrams. When normality was rejected descriptive analysis was used, calculating the median and the interquartile range (IQR). Where the distribution was compatible with normality, the mean and the standard deviation (SD) were used. For bivariable categoric variables, descriptive analysis was performed through absolute and relative frequency tables.

The possible relationship between variables was analyzed through bivariate analysis. To examine the effect of the difference in method (freehand versus dynamic guided surgery) and operator (experienced or unexperienced) and the interaction between these variables, a two independent factors analysis of variance (two-way ANOVA) was performed. Fulfillment of the test application conditions was verified through testing for normality and homogeneity of variances. This analysis was completed using graphs of the estimated averages. For statistically significant variables, the corresponding groups were compared and an estimated average was calculated for each group.

## Results

A total of 36 implants were analyzed. Descriptive and bivariate results of the main outcome variables on comparing the two implant placement systems employed (freehand and Navident®) can be observed in  Table 1. The navigation (Navident® guided surgery) group showed significantly higher accuracy for all the variables studied except entry 3D and apex depth. On the other hand, this system significantly increased the surgical procedure time.

**Table 1:**
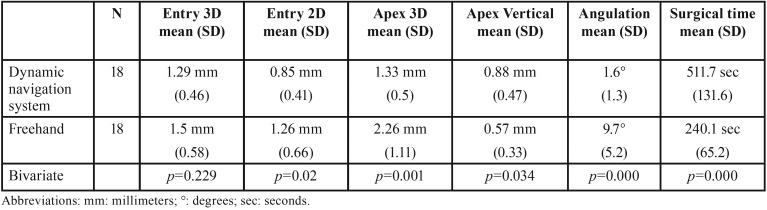
Descriptive and bivariate results of the main outcome variables for both groups.

The experienced clinician achieved more adequate angulation with the dynamic navigation system but it increased the surgery time and did not significantly improve the other parameters studied. On the other hand, it enhanced the accuracy of the novice professional significantly (entry 2D, apex 3D and angulation). All these data can be observed in  Table 2.

**Table 2:**
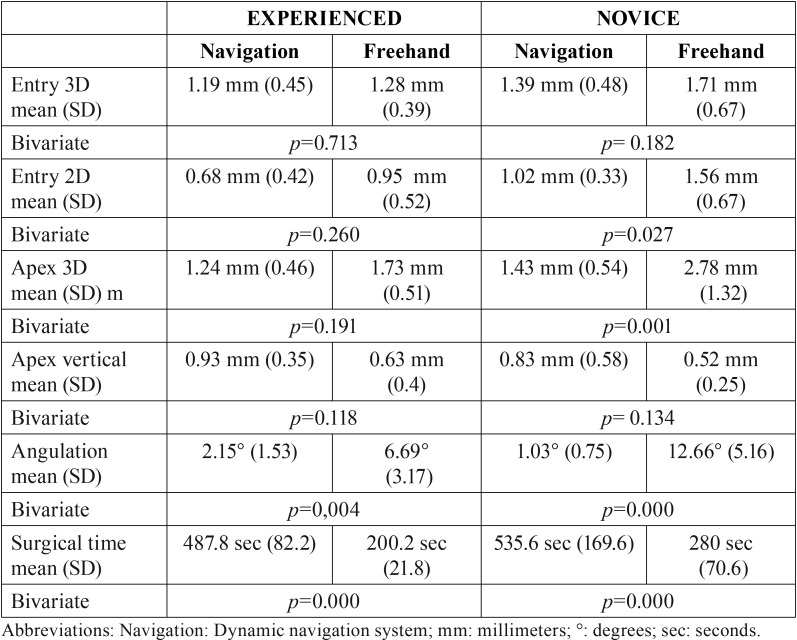
Differences between the experienced and unexperienced clinician, by group.

**Figure 3:**
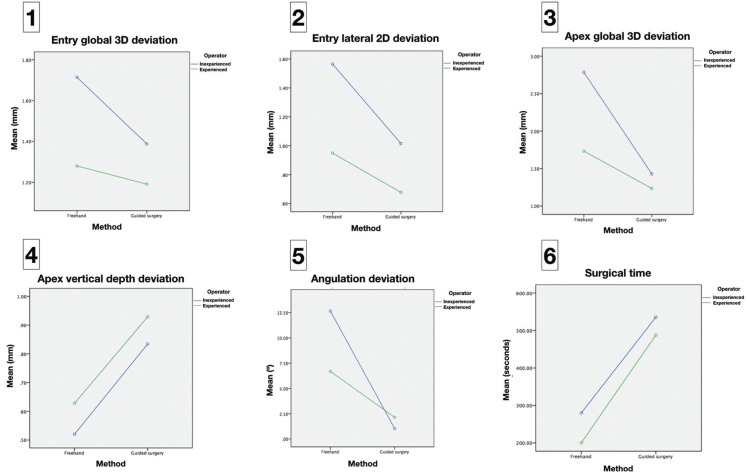
Differences between experienced and novice clinicians with the two implant placement systems.

When analyzing the results regarding the role of the surgeon’s experience, the novice professional showed a more pronounced improvement for most of the parameters studied (Fig. 3). With the freehand placement method, the experienced clinician presented significantly better results for the entry 2D (*p*=0.014), apex 3D (*p*=0.008) and angulation deviation (*p*<0.001) variables. However, these differences were negligible (*p*>0.05) when the dynamic navigation system was used.

In Figure 3 it may be seen that the navigation group showed higher accuracy in all the parameters except apical vertical depth deviation (Fig. 3) and surgical time. The latter doubled when the dynamic guided system was employed ( Table 1; Fig. 3).

## Discussion

The purpose of dynamic computer guided surgery systems in implantology is to minimize implant position deviation from the preoperative planning by employing real-time tracking of the drilling and implant insertion. Ewers *et al.* (14), after 12 years of clinical experience in this field, considered that this option provides excellent benefits, especially in delicate situations.

The usefulness of navigation relies on its high accuracy, which is particularly necessary in some specific surgical situations: (I) when anatomic structures must be taken into account and depth control is important, (II) when clinicians wish to use a flapless approach, (III) when placement requires high accuracy of angulation and spacing between implants and adjacent teeth, (IV) when implants must be placed in a tight interdental space and static guide tubes will interfere with the ideal implant position due to its size, (V) when direct visualization is expected to be difficult, such as in patients with limited mouth opening (6,15).

A meta-analysis by Jung *et al.* (9) revealed that entry point and apex accuracies are significant higher when dynamic navigation systems are used, in comparison with traditional static surgical guides. The outcomes obtained in the present *in-vitro* study are in accordance with their paper (9). However, these results should be interpreted with caution, since most of the data concerning the accuracy of dynamic systems are obtained from *in-vitro* studies using artificial models, which can lead to better results in comparison to real clinical scenarios (9). Nevertheless, several authors have shown good results in clinical studies and concluded that navigation systems are as good as static guides (11), and significantly better than freehand implant placement (11,16).

Although the results obtained with the present sample were excellent in terms of horizontal direction (entry and apex of the implant) and angulation, the outcomes related to depth accuracy were not as good as expected. The overall mean error at depth was 0.88 mm (in a range from 0mm to 1.6mm), a large discrepancy that may be considered unacceptable in anatomically compromised situations where the inferior alveolar nerve is at risk. Thus, in the authors’ opinion, a 2mm security margin should be applied to all important anatomical structures in the presurgical planning. This is an extremely important issue since neuropathic pain and sensory alterations have been described after dental implant placement (17,18).

An in-vitro study published in 2015 (7) tested the accuracy of the Navident® system and reported similar findings. Again, the results regarding depth deviation were disappointing (the deviation ranged from 0 to 3.3mm). The improvement observed in the present sample may be related to the software updates provided by the company and to small developments in the system in the past 3 years.

Other factors that might lead to incorrect positioning of the implants are CBCT scan quality, registration or planning inaccuracies, tracking system precision, acrylic splint movements, operator mistakes while following the onscreen path of drilling, and errors when overlaying the two CBCT scans (7,9,15,19).

In the opinion of the present authors the fitting of the splint is critical, and when done inappropriately might cause deviations. Future research should focus on improving the fiducial point markers and registration, since an acrylic splint can be easily deformed. Some authors report that high accuracy can only be achieved by using bone fixed fiducials because dental or mucosal supported splints can originate deviations. However, this option might increase the surgical morbidity of patients due to screw fixation (10,19).

The main advantages and drawbacks of dynamic computer guided surgery systems can be observed in  Table 3. Although these systems require a longer surgical time, entail a learning curve and are expensive, they allow a significant improvement in implant placement, especially for novice surgeons (6,9,20).

**Table 3:**
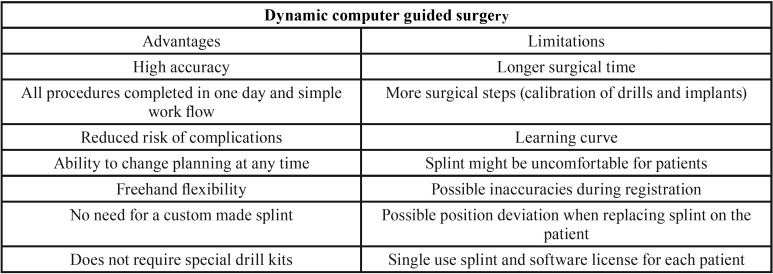
Advantages and limitations of dynamic guided surgery.

Block *et al.* (11) concluded that surgeons who had prior experience with dental navigation systems obtained better accuracy outcomes and a flat learning curve compared with professionals who were experts in implant dentistry but had no experience of navigation systems. However, the learning curve is quite fast, since after 20 cases these authors only found minimal accuracy differences between surgeons.

Although implant surgery is a common procedure in Dentistry, students consider that dental degrees offer insufficient information about implant-based treatments (21). The fact that these procedures are usually complex, involve high costs and depend on the experience of the professional is probably related to the low number of dental implant treatments performed by dental students. According to Casap *et al.* (22), final year dental students who use navigation systems improved their performance and were likely to use it in the future. This same paper showed that the learning curve was much higher for dynamic guidance than for the conventional freehand method (22).

The study design employed (*in vitro* study) might limit the generalization of the results, especially those that can be affected by clinical variables. However, the present study has high internal validity while providing control over several confounding variables that cannot be manipulated in a real clinical scenario. Indeed, the fact that all the anatomical (models, preclinical simulated patient, light conditions), surgical (drilling unit, implant system, implant length and diameter) and planning (CBCT, software used in presurgical planning) variables were identical made it possible to analyze the effect of experience on the accuracy of the systems without confounders. Another aspect that should be addressed in future research is the relation between accuracy and implant position (maxilla versus mandible; anterior versus posterior). This issue could not be analyzed in the present report due to the limited sample size and the randomization system employed.

In conclusion, dynamic computer assisted surgery systems allow more accurate implant placement in comparison with the conventional freehand method, regardless of the surgeon’s experience. However, this system seems to offer more advantages to novice professionals, since they can significantly reduce their deviations, achieving similar results to those of experienced clinicians. Since depth deviations might occur, a minimum 2 mm safety margin to relevant anatomical structures is recommended.
